# Antioxidant and hepatoprotective effects of novel heteropolysaccharide isolated from *Lobularia maritima* on CCl4‐induced liver injury in rats

**DOI:** 10.1002/fsn3.2836

**Published:** 2022-04-04

**Authors:** Anis Ben Hsouna, Mbarka Hfaiedh, Sirine Ben Slima, Walid Ben Romdhane, Boutheina Ben Akacha, Mohamed Taieb Bouterra, Wissal Dhifi, Wissem Mnif, Faical Brini, Rania Ben Saad, Riadh Ben Salah

**Affiliations:** ^1^ Department of Life Sciences Faculty of Sciences of Gafsa Gafsa Tunisia; ^2^ 117476 Laboratory of Biotechnology and Plant Improvement Centre of Biotechnology of Sfax Sfax Tunisia; ^3^ Research Unit of Active Biomolecules Valorisation Higher Institute of Applied Biology of Medenine University of Gabes Medenine Tunisia; ^4^ Laboratory of Microorganisms and Biomolecules (LMB) Center of Biotechnology of Sfax Sfax Tunisia; ^5^ Laboratory of Biotechnology and Valorisation of Bio‐GeoRessources Higher Institute of Biotechnology of Sidi Thabet Biotechpole Sidi Thabet University of Manouba Ariana Tunisia; ^6^ Department of Chemistry Faculty of Sciences and Arts in Balgarn University of Bisha Bisha Saudi Arabia; ^7^ ISBST BVBGR‐LR11ES31 Biotechpole Sidi Thabet University of Manouba Ariana Tunisia

**Keywords:** CCl_4_, genotoxicity, halophyte, *Lobularia maritima*, hepatoprotective, oxidative stress, polysaccharides

## Abstract

The aim of the present study was to investigate the extraction and the characterization of a novel heteropolysaccharide from Tunisian halophyte *Lobularia maritima* (LmPS). We were also interested in its antioxidant, anti‐inflammatory, and hepatoprotective effects on carbon tetrachloride (CCl_4_)‐induced liver injury in rats. LmPS physicochemical properties were evaluated by thin‐layer chromatography (TLC), high‐performance liquid chromatography (HPLC), thermogravimetric analysis (TGA), and UV absorption. According to TLC and HPLC results, LmPS was a heteropolysaccharide composed of glucose, galactose, and xylose. Its molecular weight was 130.62 kDa. This heteropolysaccharide was characterized by a significant antioxidant potential and was efficient against oxidative stress and CCL_4_‐induced hepatotoxicity in rat Wistar models (*n* = 8) treated with a single dose of LmPS 250 mg/kg of body weight. This was evidenced by a significant increase in serum marker enzymes specially aspartate transaminase (AST), alanine transaminase (ALT), alkaline phosphatase (ALP), and lactate dehydrogenase (LDH). The cytokines released after stimulation of rats with LmPS showed high anti‐inflammatory profiles with an increased rate of interleukine‐10 (IL‐10) with 0.03 pg/mL compared to animals treated only with CCl_4_. On the contrary, we noticed a decrease of the other cytokines (tumor necrosis factor α: TNF‐α, interleukine‐6: IL‐6, transforming growth factor beta 1: TGF‐β1) with average concentration values of <0.2, 0.1, and 0.04 pg/mL, respectively. Besides, histopathological examinations revealed that CCl_4_ causes acute liver damage, characterized by extensive hepatocellular necrosis, vacuolization, and inflammatory cell infiltration, as well as DNA fragmentation. LmPS administration at a dose of 250 mg/kg resulted in a significant hepatoprotection, evidenced by a reduction of CCl_4_‐induced oxidative damage for all tested markers. These findings eagerly confirmed that LmPS was effective in the protection against CCl_4_‐induced hepatotoxicity and genotoxicity. It, therefore, suggested a potential therapeutic use of this polysaccharide as an alternative medicine for patients with acute liver diseases.

## Graphical abstract

The current study was designed for the extraction and characterization of a novel heteropolysaccharide from Tunisian *L. maritima* (LmPS). In the experiment, the physicochemical properties of LmPS were evaluated by TLC and HPLC. In vivo, the antioxidant potential and the efficacy of LmPS against oxidative stress and CCL4 were evaluated using serum marker enzymes (AST, ALT, ALP, LDH). The anti‐inflammatory profiles were also evaluated using interleukine‐10 (IL‐10) and other cytokine levels (TNF‐α, IL‐6, and TGF‐β1). A histopathological diagnosis was needed for the detection of liver damage.

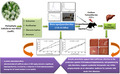

## 1 INTRODUCTION

Environmental pollution and some toxic chemicals like carbon tetrachloride (CCl_4_) lead to toxic radical species genesis can cause lipid peroxidation (LPO) and damage to the organism cells. This exposure can come from the drinking water, air, foodstuffs, soil, and some industrial places where industrial contamination have occurred, or these chemicals are applied (Ekpo et al., 2020). In the biological system, CCl_4_‐induced toxicity begins with the bio‐activation of CCl_4_ by the hepatic cytochrome P‐450 oxygenase system through reductive dehalogenation, resulting in the formation of trichloromethyl radical (CCl_3_*). This radical species, first formed as an unreactive metabolite of CCl_4_, is highly reactive with molecular oxygen. This leads to the production of trichloromethylperoxyl radical (CCl3OO*) (Asai et al., 2005). CCl_3_OO* then binds covalently to cellular macromolecules in the cell membrane, causing functional and morphological changes that lead to degeneration of fats, fibrosis, cirrhosis, cell death, cancer, and the necrosis of hepatocytes (Yousefi‐Manesh et al., 2020). Hence, CCl_4_ was widely used as a known chemical hepatotoxin for the induction of hepatotoxicity in experimental animals such as rats (Zhang et al., 2017). Oxidative stress results in an important depletion of intrinsic antioxidants and arise of LPO (Jadeja et al., 2015). For redox balance, hepatocytes use first‐line antioxidant defense proteins and other molecules to improve this effect. Besides, hepatic oxidative stress control using antioxidants is a key factor in protecting against liver diseases or accelerates recovery of damaged hepatocytes (Jadeja et al., 2015; Mirhosseini, 2017). Although liver damage may lead to death worldwide, therapeutic interventions targeted at protecting the hepatocytes from damage or repair of damaged hepatocytes are largely limited.

Recently, advancement in scientific research has paved the way for the isolation of bioactive phytochemicals with pharmacological effects, which are now used as potential therapeutic agents (Ben Hsouna et al., 2011).

Polysaccharide is renowned for its wide pharmacological effects, such as antioxidant, inhibition of platelet aggregation, reduction of blood cholesterol concentrations, alleviation of heart disease, and blood glucose levels, and it has also been reported to prevent liver LPO (Patil et al., 2018).

Great interest has been given to mushrooms that contain several important biomolecules such as lectins, proteases, and polysaccharides (Palanisamy et al., 2017). These molecules are characterized by numerous bioactivities and important therapeutic capacities (Zheng et al., 2016). Bioactive polysaccharides are contained in several organisms such as plants, bacteria, fungi, and algae (Yang & Zhang, 2009).

*Lobularia maritima* (*Alyssum maritimum*, Brassicaceae) has the common name of sweet alyssum or sweet Alison. It is a halophyte endemic in Tunisia and is salinity tolerant with a value up to 400 Mm NaCl (Ben Hsouna et al., 2020). This plant is reputed for antiscorbutic and diuretic (Ben Hsouna et al., 2020) as well as for its astringent properties (Ben Hsouna et al., 2022).

No data are available concerning extraction and characterization of *L*. *maritima* polysaccharides (LmPS) from this Tunisian halophyte and its effects on the hepatoprotectives effects on CCl_4_‐induced hepatotoxicity in rats. Considering the promising properties of *L*. *maritima,* the present study aims to explore the extraction, characterization, structure, and antioxidant and anti‐inflammatory activities of LmPS. The effects of LmPS on CCl_4_‐induced hepatotoxicity in rats are also studied.

## 2 MATERIAL AND METHODS

### 2.1 LmPS extraction

The aerial parts of *L. maritima* were collected in March 2020 from the Chebba region (Mahdia, Tunisia, latitude 35.23°, longitude 11.11°). The aerial parts were air‐dried in shade at 25°C for 2 weeks, pulverized with a blender for 15 min, and stored in dry, dark, controlled conditions (25°C) at room temperature. *Lobularia maritima* powder was pre‐extracted with 95% ethanol at room temperature to remove pigments. The dry residue was extracted twice with 20 volumes of deionized water at 90°C while tiring for 4 h. The extract was combined and filtered, and filtrates were then evaporated under vacuum. The concentrated liquid was precipitated with 95% (v/v) ethanol at 4°C during 24 h and then centrifuged (4500 *g*) for 15 min using a refrigerated centrifuge. Afterward, the precipitate was dried at 60°C and stored at 4°C until further use. The yield was calculated concerning the initial mass (g) of *L. maritima* powder.

### 2.2 In vitro antioxidant assays of LmPS

#### 2.2.1 DPPH scavenging activity

LmPS activity was evaluated by bleaching the DPPH methanolic solution, following the methods described by Sarker et al. (2006). We carried on an extraction of 100 mg of LmPS in 1 ml of distilled water. The content was filtered. Various concentrations (0–300 µg/ml) of the supernatant were added to 1 ml of a 0.1 mM methanolic solution of DPPH and kept for 45 min at 27°C in darkness. Optical density (OD) changes were measured at 517 nm with methanol as blank and Catechin as standard. The scavenging effect was calculated as follows:

$$\begin{array}{c} \%\;\text{Inhibition of DPPH radical}\\ =\left[\left(\text{Control OD}‐\text{Sample OD}\right)/\text{Control OD}\right]\times 100 \end{array}$$

The effective concentration (IC_50_) was defined as the concentration (in µg/mL) of the compound required to scavenge 50% of DPPH.

#### 2.2.2 β‐Carotene–linoleic acid bleaching assay

This potential was evaluated by using the β‐carotene bleaching method (Miraliakbari & Shahidi, 2008) with minor modifications. Seven hundred fifty microliter of a β‐carotene chloroform solution, 33 μl of linoleic acid, and 225 mg of Tween 40 were mixed. The solvent removal was done with a rotary evaporator. Then oxygenated distilled water was added, and mixture emulsification was carried on in a sonicator. Aliquots were transferred into stopper test tubes containing 1 ml of samples dissolved in various concentrations of DMSO. OD was read at 470 nm immediately (*t* = 0) and at the end of the time (*t* = 120) for all samples. The blank was a second emulsion which is a mixture of oxygenated water, 22 μl of linoleic acid, and 150 mg of Tween 40 and the standard was Catechin. The test was carried out in triplicate. The percentage of inhibition was calculated as follows:

$$\begin{array}{c} \%\;\text{Inhibition}\\ =\left[\left({\text{Sample OD}}_{\left(120\right)}‐{\text{Control OD}}_{\left(120\right)}\right)/\left({\text{Control OD}}_{\left(0\right)}‐{\text{Control OD}}_{\left(120\right)}\right)\right]\;\times 100 \end{array}$$

### 2.3 Chemical analysis of LmPS

Characterization of LmPS was expressed in terms of moisture, ash, fat, carbohydrate, protein, and color. The moisture and ash content were determined according to the standard of the Association of Official Analytical Chemists (AOAC) International (Horwitz, 2000).

### 2.4 LmPS spectroscopic analysis

#### 2.4.1 LmPS average molecular weight

The average molecular weight (M_w_) was assayed using the method of Bayar et al. (2016).

#### 2.4.2 UV absorption peak detection of LmPS

LmPS was dissolved in distilled water to a final concentration of 0.05%. The UV absorption spectrum of the sample was recorded in the wavelength range of 200–800 nm (He et al., 2015).

#### 2.4.3 TLC analysis of LmPS

The TLC analysis of the hydrolysate was conducted on a silica gel plate (Merck, Germany). We used a mixture of chloroform/acetic acid/water (6:7:1, v/v/v) as mobile phase.

#### 2.4.4 HPLC analysis of LmPS

LmPS were hydrolyzed in trifluoroacetic acid (4 M, TFA) at 100°C for 12 h. Monosaccharide composition was analyzed by HPLC using an Aminex HPX‐87H column with a mobile phase of 0.001 NH_2_SO_4_, column temperature of 60°C, and a flow rate of 0.5 ml/min.

#### 2.4.5 Thermogravimetric analysis of LmPS

Thermogravimetric analysis (TGA) was tested using thermogravimetric analyzer (Mettler Toledo TGA/SDTA 8951E). Approximately 5 mg of sample was introduced into the sample pan and heated from 30 to 400°C under nitrogen atmosphere. The gas flow rate was 40 ml/min.

### 2.5 Acute toxicity study

The acute toxicity study of LmPS was evaluated according to the method of OECD (G, 2000). The rats had fasted overnight with free access to water. A single dose of LmPS 2 g/kg of body weight was orally gavaged to two groups LmPS (*n* = 8) of male Wistar rats. All the experimental animals were maintained under close monitoring to observe the signs of toxicity and mortality if any, twice daily for 20 days.

### 2.6 Hepatoprotective effect of LmPS on CCl4‐induced liver injury

#### 2.6.1 Experimental design and protocol

Thirty‐two male Wistar albino rats from Pasteur Institute (Tunisia), with relative weights ranging from 180 to 220 g, were obtained after approval of the Institutional Animal Ethics Committee of Tunisian University.

Standard laboratory conditions (24 ± 2°C, 12/12 light/dark cycle) were adopted for all the animals fed with a standard pellet diet and allowed for free access to drinking water, given ad libitum during 15 days of the experimental period. Before starting the experiment, all the animals were allowed to acclimatize for laboratory conditions for a week. In a multiple‐dose pretreatment experiment, LmPS was administered at 250 mg/kg by intraperitoneal injection (i.p.).

The male rats were randomly divided into four groups (8 rats in each group):

- Group I: control animals received the vehicle (olive oil, 1 ml/kg orally) on day 14.
- Group II: hepatoxicity model received a single dose of CCl_4_ in olive oil (1 ml/kg, i.p.) on the 14th day (Ben Hsouna, Dhibi, Dhifi, Ben Saad, Brini, et al., 2019; Ben Hsouna, Dhibi, Dhifi, Mnif, et al., 2019; Ben Hsouna, Gargouri, Dhifi, Ben Saad, Sayahi, et al., 2019) on the 14th day.
- Group III: received LmPS + CCl_4_, was pretreated with LmPS, and intoxicated with CCl_4_ on the 14th day. The CCl_4_ dose was selected according to the chronic oral reference dose as recommended for CCl_4_ (Ben Hsouna, Dhibi, Dhifi, Mnif, et al., 2019; Ben Hsouna, Gargouri, Dhifi, Ben Saad, Sayahi, et al., 2019; Ben Hsouna, Gargouri, Dhifi, & Saibi, 2019).
- Group IV: received LmPS (250 mg/kg BW) daily by i.p. injection for 15 days, a daily i.p. injection of LmPS at 250 mg/kg of body weight, and distilled water as a sole beverage (Ben Hsouna, Dhibi, Dhifi, Ben Saad, Brini, et al., 2019; Ben Hsouna, Dhibi, Dhifi, Mnif, et al., 2019).

The animals were sacrificed on day 15 by cervical decapitation. Blood samples were collected directly. Serums were transferred into 0.5 ml vials, then kept at −80°C for the further biochemical tests. The excised liver was minced with ice‐cold saline. The supernatant (S1) was frozen at −30°C in aliquots until analysis.

#### 2.6.2 Liver function test

- Estimation of hepatic aspartate transaminase (AST): Activity of hepatic AST was measured spectrophotometrically using commercially available kits, according to the manufacturer's instructions. Briefly, plasma (100 μl) was added to 1 ml of prewarmed working solution (as provided in the kit) and absorbances measured at 340 nm. The first reading was taken at 1 min followed by three consecutive readings at 30 s interval. The activity of AST was calculated per minute and expressed as IU/L.
- Estimation of hepatic alanine transaminase (ALT): Activity of hepatic ALT was measured spectrophotometrically using commercially available kits, according to the manufacturer's instructions. Briefly, a working solution (as provided in the kit) was prepared by mixing reagents R1 and R2 in the ratio 4:1. Plasma (50 μl) was added to 1 ml of prewarmed working solution and absorbances measured at 340 nm, the first reading being at 1 min followed by three consecutive readings at 30 s interval. The activity of ALT was calculated per minute and expressed as IU/L.
- Estimation of hepatic alkaline phosphatase (ALP): Activity of hepatic ALP was measured spectrophotometrically using commercially available kits, according to the manufacturer's instructions. Briefly, a substrate tablet was reconstituted in the diluent and the working solution was prepared. Plasma (20 μl) was added to 1 ml of prewarmed working solution and absorbances measured at 450 nm, the first reading being at 1 min followed by three more consecutive readings at 30 s interval. The activity of ALP was calculated and expressed as IU/L.
- Estimation of hepatic lactate dehydrogenase (LDH): Activity of hepatic LDH was measured spectrophotometrically using commercially available kits, according to the manufacturer's instructions. Briefly, the working solution was prepared by mixing L2 (Starter Reagent) and L1 (Buffer Reagent). One milliliter of working solution was incubated at 30°C for 1 min, followed by addition of 50 μl plasma. The first reading was taken at 1 min by measuring absorbances at 340 nm followed by three more readings at 30 s interval. The activity of LDH was calculated per minute and expressed as U/L.

#### 2.6.3 LPO status

Malondialdehyde (MDA) levels were determined according to the method of Ohkawa et al. (1979) at 532 nm. MDA, a product of LPO, was used as a standard. Briefly, tissues were homogenized (10% w/v) in PBS containing butylated hydroxytoluene (0.01%) to prevent further oxidation. After centrifugation (12,000 *g* for 15 min, 4°C), the supernatant (300 μl) was added to a mixture (600 μl) of TCA (15%), TBA (0.375%), and HCl (0.25 N). It was agitated at 100°C for 30 min in a water bath, cooled, and the resultant pink colored complex [TBA–MDA complex] was extracted by butanol and pyridine (15:1 v/v). The amount of MDA was calculated using an extinction coefficient of 156 mM^−1^ cm^−1^ and expressed in nmoles MDA/mg protein.

Protein concentration in tissue homogenates was estimated using bovine serum albumin as standard (Lowry et al., 1951).

#### 2.6.4 Antioxidant enzymes assay

Superoxide dismutase (SOD), catalase (CAT), and glutathione peroxidase (GPx) were determined using commercially available kits based on spectrophotometric analysis by ELISA reader (StatFax 3000).

- Estimation of hepatic SOD activity: Briefly, liver homogenates (10% w/v in Tris‐HCl buffer, 50 mM, and pH 7.5) containing diethylene triamine penta acetic acid (2 mM) were centrifuged (10,000 *g*, 30 min, 4°C). To the supernatant, 125 μl of ethanol and 75 μl of chloroform were added, vortexed for 5–7 min, and after centrifugation (13,000 *g*, 15 min, 4°C) the upper layer (10 μl) was incubated with a reaction mixture, containing pyrogallol solution (2 mM, 0.4 ml), and distilled water (1.6 ml). The auto‐oxidation of pyrogallol was measured every 30 s for 3 min, by measuring the increase in absorbance at 420 nm. One unit of SOD activity was defined as the amount of enzyme required to inhibit the rate of pyrogallol auto‐oxidation by 50% and expressed as unit/mg of protein.
- Estimation of CAT activity: Liver tissue (100 mg) was homogenized in 500 μl of ice‐cold phosphate buffer (0.075 M, pH 7.4) and CAT activity determined spectrophotometrically using H_2_O_2_ (30%) as the substrate in phosphate buffer and expressed as unit/mg protein. Briefly, lysates (10 μl) were added to 3 ml of H_2_O_2_‐phosphate buffer and absorbances measured at 420 nm every 30 s for 3 min against a blank containing the lysate in phosphate buffer. One unit of CAT activity was defined as the amount of enzyme that degrades 1 μM H_2_O_2_ per minute and expressed as unit/mg of protein.
- Estimation of GPx activity: GPx activity in liver cytosol was determined by NADPH oxidation using a coupled reaction system consisting of reduced glutathione (GSH), glutathione reductase (GR), and H_2_O_2_. Homogenates (10% w/v) were prepared in phosphate buffer (2.4 mM, pH 7.0) containing sodium azide (0.24 mM) and EDTA (0.28 mM). After centrifugation (13,000 *g* for 10 min), the supernatant (100 μl) was incubated for 10 min with a reaction mixture (800 μl phosphate buffer containing 2.5 mM EDTA and 2.5 mM NaN3, 10 mM GSH, 2.5 mM NADPH, and 2.4 units of GR). Following the addition of 12 mM H_2_O_2_ (100 μl), the decrease in NADPH absorbance at 340 nm was measured every 30 s for 3 min. The enzyme activity was expressed as l M of NADPH utilized min^−1^ mg^−1^ of protein using the extinction coefficient of NADPH at 340 nm being 6200 M^−1^ cm^−1^.

#### 2.6.5 Histopathological evaluation

For histological examination, liver tissue samples were fixed in 10% formalin for at least 24 h. After fixation, tissue samples dehydrated using ascending concentrations of ethanol (70, 90, 95, and 100%), cleared in xylene, and embedded in the paraffin were cut at 4 μm sections for histopathological evaluation. Tissue preparations were observed and microphotographed.

#### 2.6.6 Enzyme‐linked immunosorbent assay (ELISA)

After collection, we used the supernatants (S1) for the evaluation of some immunological parameters by ELISA (Ben Hsouna et al., 2021).

#### 2.6.7 Qualitative DNA fragmentation assay by agarose gel electrophoresis

Mammalian tissues (liver) were lysed with a chaotropic salt‐containing buffer to ensure denaturation of macromolecules. Qualitative DNA fragmentation DNA was done according to the method of Hfaiedh et al. (2019).

### 2.7 Statistical analysis

The results were analyzed using Graph Pad Prism (version 5, Graph Pad Software Inc., La Jolla, CA, USA). Results for each group are presented as mean ± standard deviation (*SD*) for eight rats. One‐way ANOVA, post hoc test, and Fisher's least significant difference (LSD) test were used to evaluate the statistical significance of the differences between LmPS groups.

## 3 RESULTS

### 3.1 Physicochemical analysis of LmPS

The chemical composition of the LmPS is presented in Table 1. The obtained LmPS amount was calculated at 8.5 ± 0.89 (w/w) according to *L. maritima* dry weight. The results proved that total sugars were the most interesting part (84.85 ± 0.56%) of the extract. The ash and fat were estimated at 7.43% and 0.1%, respectively. Additionally, the sample was characterized by relatively low moisture (7.02%). Interestingly, LmPS were characterized by an important light value (L* = 56.92) and a slight degree of redness (2.19). This is in concordance with data reported by Ben Slima et al. (2018).

**TABLE 1:** Chemical composition of the LmPS

Parameters	LmPS
Yield (%)	8.5 ± 0.89
Moisture (%)	7.02 ± 0.09
Ash (%)	7.43 ± 0.25
Protein (%)	0.6 ± 0.04
Fat (%)	0.1 ± 0.01
Carbohydrates (%)	84.85 ± 0.56
Color	
a*	2.19 ± 0.01
b*	17.20 ± 0.03
L*	56.92 ± 0.01

### 3.2 Average molecular weight

**FIGURE 1:**
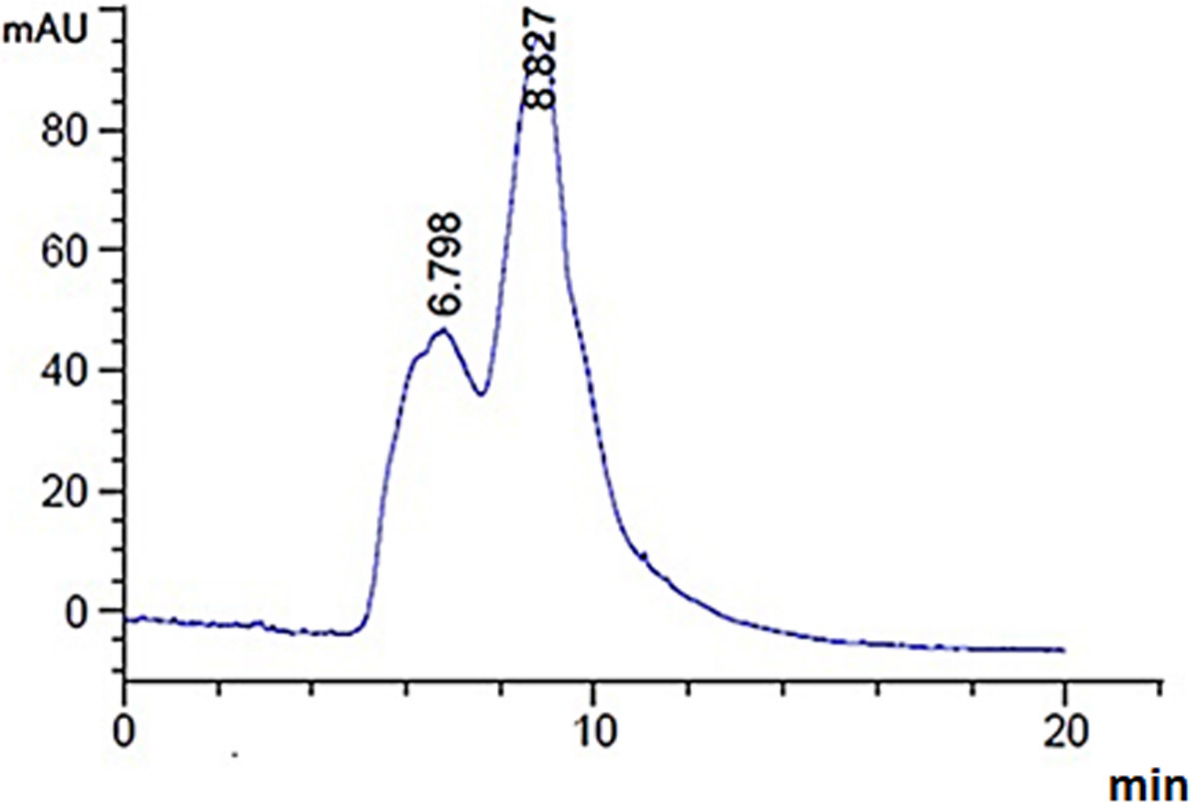
Assessment of LmPS average molecular weight

According to Figure 1, LmPS recorded two peaks at 6.798 and 8.827 min, and the major average molecular weight were estimated to be 130.62 kDa.

### 3.3 UV‐visible spectroscopy

**FIGURE 2:**
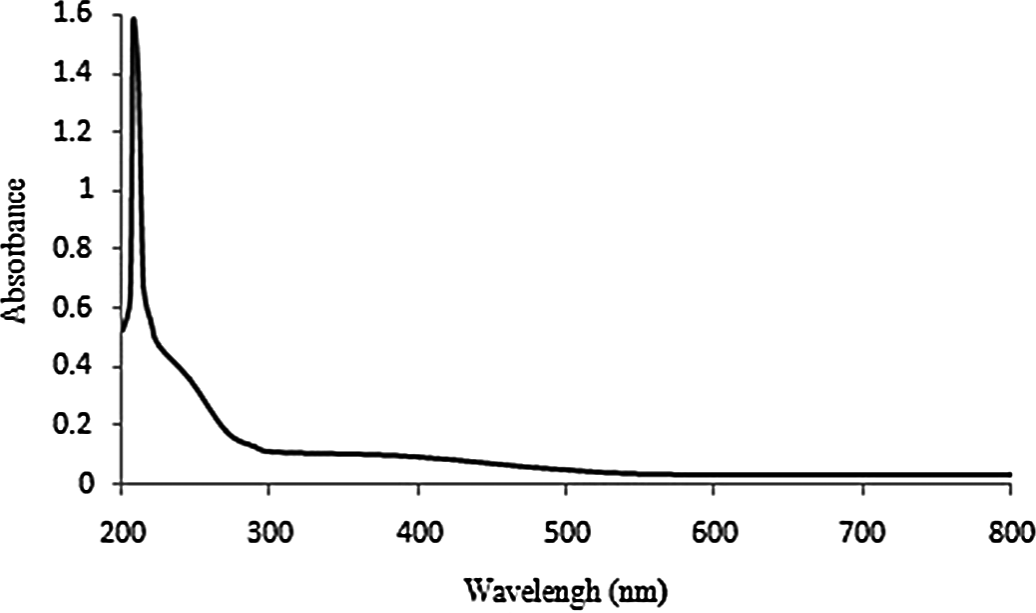
Scan of LmPS within the wavelength range of 200–800 nm

UV‐visible spectrum was recorded on 200–800 nm range in the different levels of absorbance peaks. The range from 200 to 220 nm, confirm the presence of polysaccharides (Ben Slima et al., 2018) as shown in Figure 2.

### 3.4 Infrared spectrum analysis

The structure and the functional groups of LmPS were characterized with the FTIR spectroscopy at 500–400 cm^−1^. We noted typical peaks at 1,558; 1,303; 1,035; 950; and 845 cm^−1^. LmPS broadly exhibited OH‐structuring variation at 3,643 cm^−1^ indicating the intermolecular and intramolecular hydrogen bands formation. The band at 2,881 cm^−1^ may be assigned to the structuring variation of OH‐groups. An additional band was observed at 1,558 cm^−1^ due to the bound water (Ben Slima et al., 2018). CO‐group absorb at 1,303 cm^−1^. Weak absorption below 1,000 cm^−1^ revealed the visible band's presence and/or possible monosaccharide bounds.

### 3.5 TGA

**FIGURE 3:**
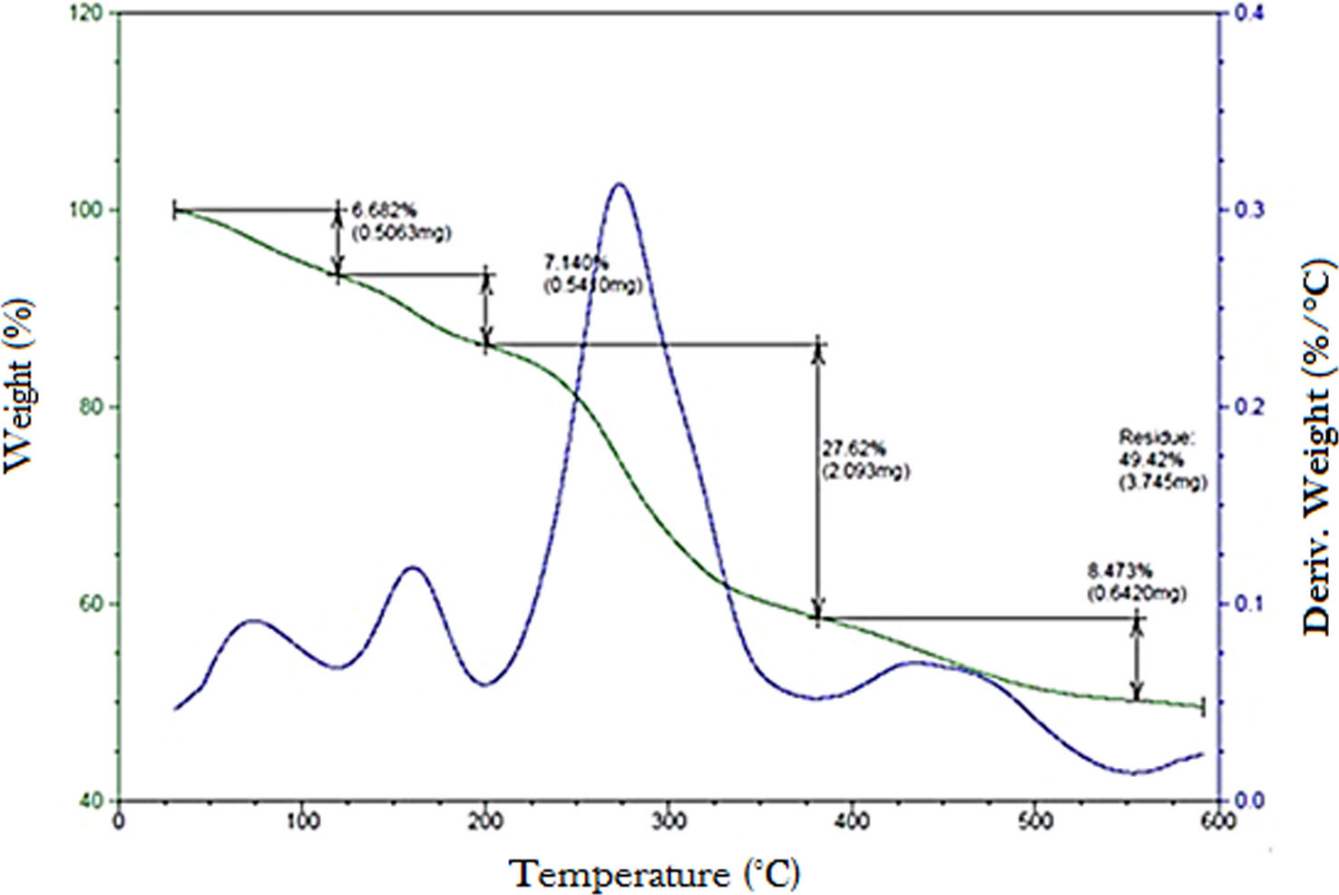
TGA of LmPS

TGA was used to evaluate polysaccharide stability at high temperatures. In fact, it could provide the mass change of sample related with decomposition, dehydration, and oxidation. Consequently, these modifications affected the structure and the quality of final products (Nawrocka et al., 2017). The results showed four mass loss events for LmPS (Figure 3). Three mass losses were noted at 30.19; 119.31; and 243°C. This fact could be due to biopolymer water absorption or due to desorption (Kittur et al., 2002). Polysaccharide thermal decomposition could explain the weight loss even at 381.41°C, resulting 8.47% of weight loss, may be attributed to degradation reactions (Xie et al., 2013).

### 3.6 Monosaccharide composition analysis by TLC

**FIGURE 4:**
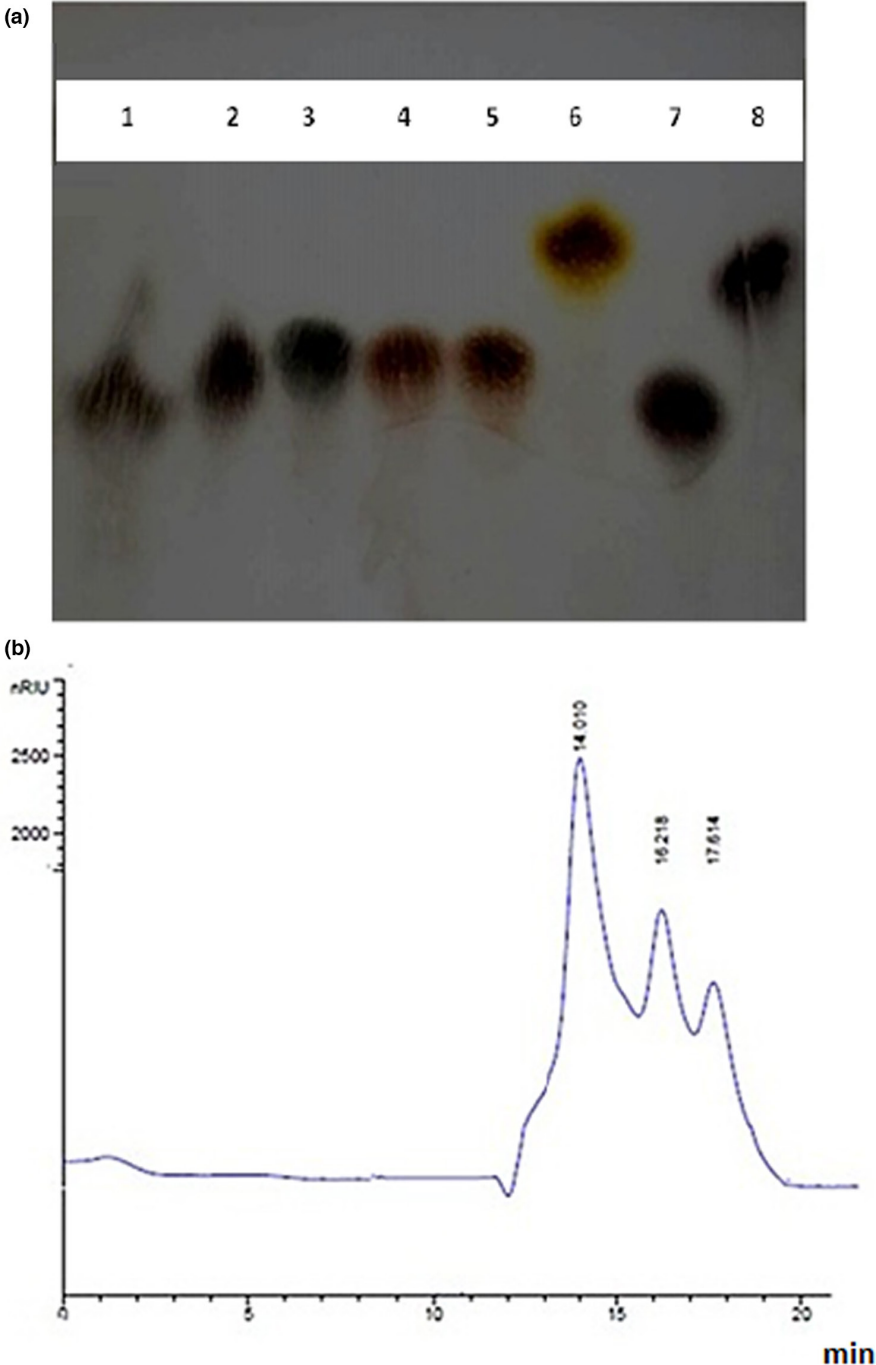
(a) TLC analysis of LmPS hydrolyzed by TFA. 1: PS hydrolyzed by TFA; 2: Glucose; 3: Fructose; 4: Mannose; 5: Arabinose; 6: Rhamnose; 7: Galactose; 8: Xylose; (b) HPLC analysis of LmPS hydrolyzed by TFA

TLC LmPS results revealed three plugs with retention factor of 0.55, 0.58, and 0.78 corresponding to glucose, galactose, and xylose, respectively (Figure 4a).

### 3.7 Monosaccharide composition analysis by HPLC

As indicated in Figure 4b, the LmPS showed three peaks: the major peak (RT = 14.01 min) corresponding to glucose and two minor peaks with retention time of 16.21 and 17.61 min corresponding to galactose and xylose, respectively. These results confirmed the data obtained by TLC.

### 3.8 In vitro antioxidant capacities of crude polysaccharide LmPS

**FIGURE 5:**
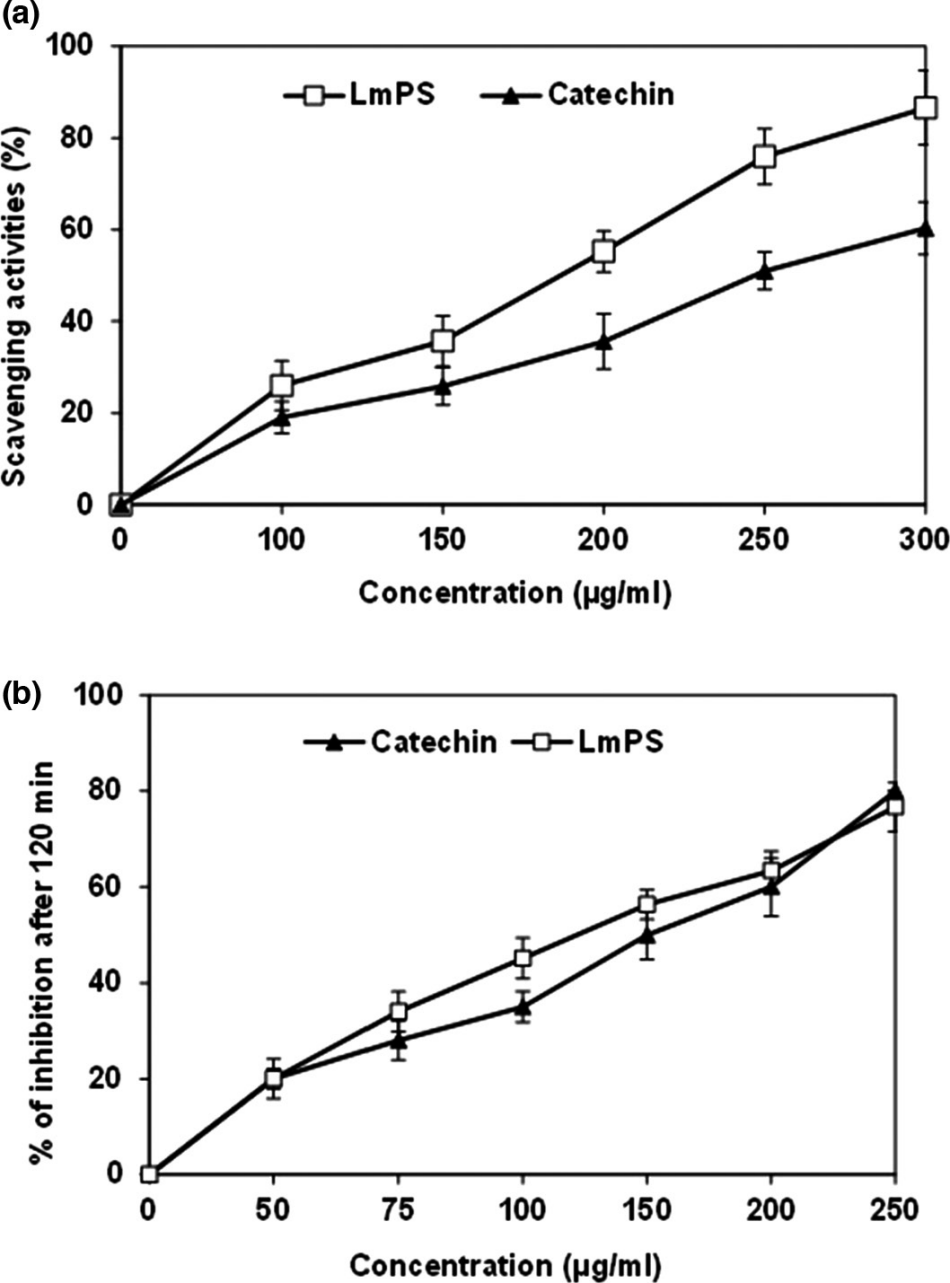
(a) Scavenger effect of *LmPS* at different concentrations (µg/ml); (b) Antioxidant activities of *LmPS* at different concentrations (µg/mL)

According to Figure 5a, we noted an increase in DPPH scavenging activities. This was proportional to the rise of LmPS concentrations. At high concentrations (300 µg/ml), the scavenging activities of LmPS against DPPH reached 90%, these percentages were higher than those obtained with catechin at the same dose. Further, LmPS recorded EC_50_ of 0.2 mg/ml which was lower than catechin (0.25 mg/ml) used as standard.

Moreover, Figure 5a showed β‐carotene bleaching after the addition of the LmPS fraction and Catechin. As found for the previous test, this antioxidant activity was proportional to the dose. The LmPS had an important antioxidant activity (IC_50_ = 130 µg/ml) close to that Catechin one (50 µg/ml).

### 3.9 Hepatoprotective effect of LmPS on CCl_4_‐induced liver dysfunction

#### 3.9.1 Acute toxicity studies

The LmPS treated rats did not show any toxicity. LmPS was nontoxic and safe. The LD_50_ was above 250 mg/kg.

#### 3.9.2 Serum markers

As shown in Table 2, there was a significant increase in AST, ALT, ALP, and LDH activities in CCl_4_‐treated animals compared to the control group that indicated a toxic effect on liver toxicity. Interestingly, a dose of LmPS 250 mg/kg of body weight decreases CCl_4_‐induced liver function markers.

**TABLE 2:** Effects of CCl_4_, LmPS, and their combination (LmPS/CCl_4_) on hepatic serum markers of control and experimental rats

Treatment	AST	ALT	ALP	LDH
C	160 ± 3.75	40.55 ± 7.6	60.25 ± 7.4	890 ± 85
CCl_4_	230.9 ± 14.2 ^***^	93.33 ± 10.32^**^	95.33 ± 10.86^**^	1,350 ± 135^***^
LmPS/CCl_4_	156 ± 5.7^##^	37.7 ± 9.3^##^	58.4 ± 6.8^###^	885.55 ± 78.33^###^
LmPS	157 ± 5.9	39 ± 3.6	60.33 ± 1.6	887 ± 90.33

Note

Values are mean ± SEM for eight rats in each group. CCl_4_, LmPS, and LmPS/CCl_4_‐treated groups versus control group; ^**,##^ *p* < .01, ^***,###^ *p* < .001, CCl_4_ group versus (LmPS/CCl_4_).

#### 3.9.3 Oxidative stress analysis

**FIGURE 6:**
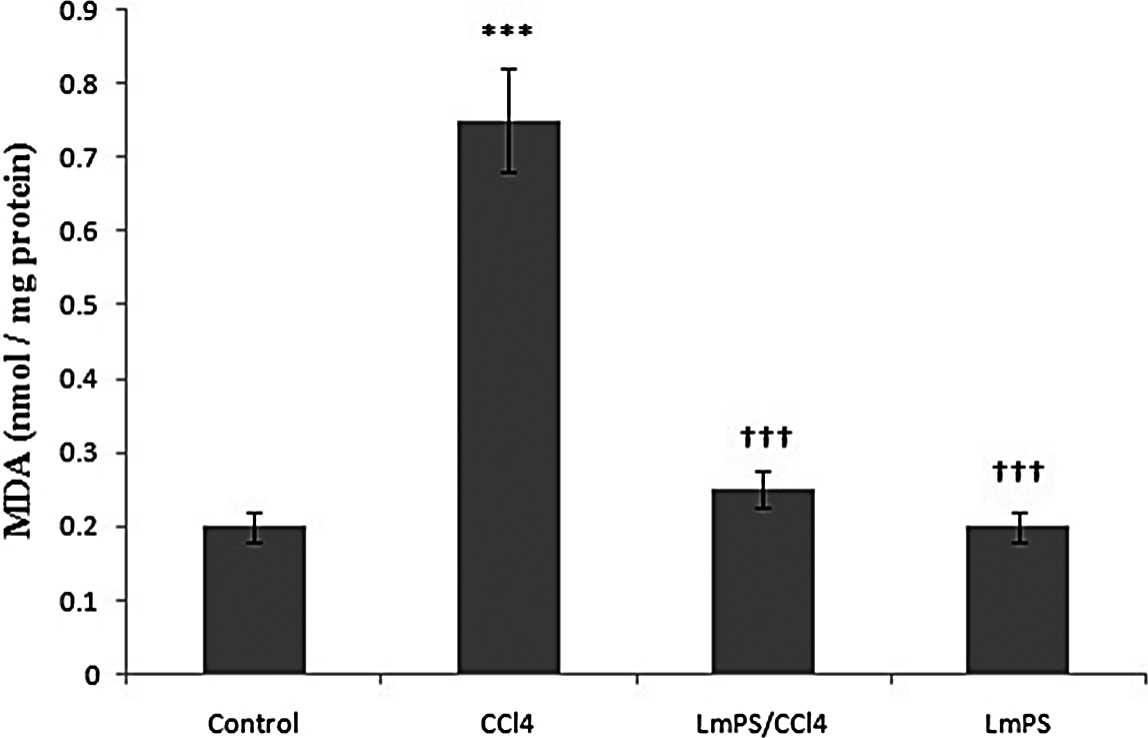
Effects of CCl_4_, LmPS (Lin) and their combination on hepatic MDA of control and experimental rats. Data show mean ± SEM values of three independent experiments. ^***^ *p* < .001 indicates significant differences compared to CCl_4_ group versus control. ^†††^ *p *< .001 indicates significant differences compared to LmPS group versus CCl_4_

MDA level was widely used to evaluate the importance of free radical‐mediated LPO injury. We determined MDA levels in the liver, and the results are shown in Figure 6. The hepatic MDA content was significantly (*p* < .001) increased (0.75 ± 0.07 nmol/mg protein) in CCl_4_‐treated animals. Prior oral administration of LmPS along with CCl_4_ significantly lowered the levels of MDA content in liver (0.25 ± 0.025 nmol/mg protein) when compared to CCl_4_‐treated rats.

Control and experimental rats’ antioxidative enzyme levels are shown in Table 3. A significant decrease (*p* < .001) in SOD, CAT, and GPx activities was noticed in rats treated only with CCl_4_ (11.93 ± 0.258 units/mg protein, 290.66 ± 14.04 µmol H_2_O_2_/mg protein, 4.85 ± 0.409 µmol GSH/min/mg protein, respectively) by reference to normal rats (22.5 ± 2.17 units/mg protein, 445.33 ± 16.59 µmol H_2_O_2_/mg protein, 11.75 ± 0.839 µmol GSH/min/mg protein, respectively). However, tissue antioxidant levels of LmPS‐supplemented animals were similar to control ones.

**TABLE 3:** Effects of CCl_4_, LmPS, and their combination (LmPS/CCl_4_) on the enzymatic antioxidant activities in liver of control and experimental rats

Treatment	SOD (Units/mg protein)	CAT (µmol H_2_O_2_/mg protein)	GPx (µmol GSH/min/mg protein)
C	22.5 ± 2.17	445.33 ± 16.59	11.75 ± 0.839
CCl_4_	11.93 ± 0.258^**^	290.66 ± 14.04^***^	4.85 ± 0.409^***^
LmPS/CCl_4_	20.53 ± 2.11^##^	438.77 ± 15.25^##^	9.25 ± 0.378^###^
LmPS	21.28 ± 0.456	440.25 ± 10.18	10.33 ± 0.4

Note

Values are mean ± SEM for eight rats in each group. CCl_4_, LmPS, and LmPS/CCl_4_‐treated groups versus control group; ^**,##^ *p* < .01, ^***,###^ *p* < .001, CCl_4_ group versus (LmPS/CCl_4_).

#### 3.9.4 Histopathological evaluation

**FIGURE 7:**
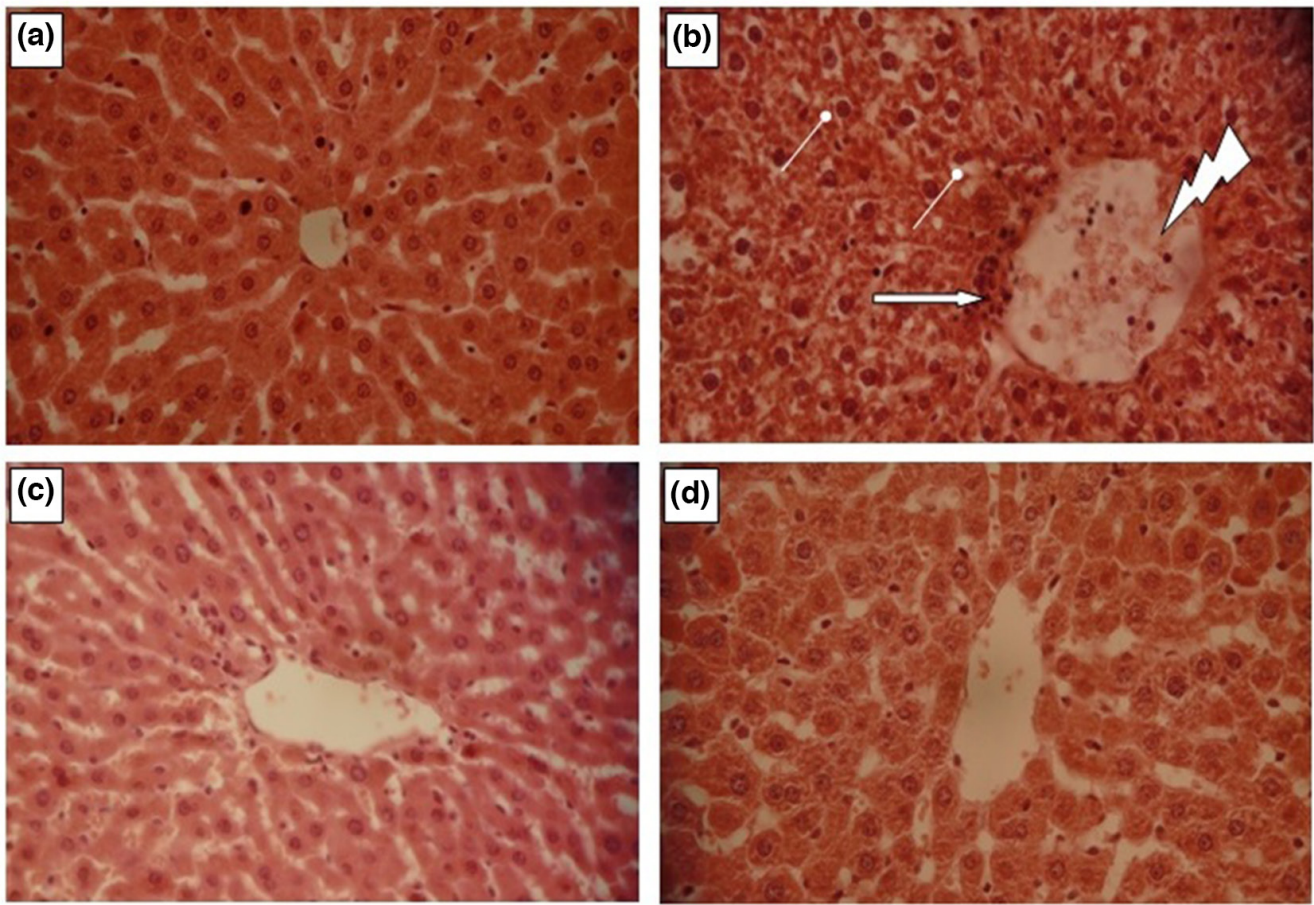
Histopathological observation of liver tissues in both control and experimental animals (a). Group 1 served as control. (b) Group 2 rats were induced hepatic damage by daily intraperitoneal injection of CCl_4_ (1 ml/kg in 1% olive oil. i.p.) for 14 day. Arrow indicates leukocyte inflammatory cells. Congested central veins. Hepatocyte vacuolization. (c) Group 4 rats were pretreated daily with LmPS (250 mg/kg BW) for 14 days and then intoxicated with CCl_4_ on the 14th day (1 mg/kg BW CCl_4_). (d) Group 3 rats were daily received LmPS (250 mg/kg BW) for 14 days. Optic microscopy: HE (×400). Scale bars = 100 µm

Liver histopathological evaluation was done using a light microscope observation (Figure 7a‒d). A usual histology of liver with a central vein and hepatocytes were apparent in the control group (Figure 7c). Conversely, numerous visible damages were induced by CCl_4_ such as necrosis associated with neutrophilic infiltration (Figure 7b). These damages were significantly reduced by LmPS administration, and no necrosis and degenerations were noticed (Figure 7d). These results corroborate with those relative to serum biochemical parameters and oxidative stress markers.

### 3.10 Anti‐inflammatory effects of LmPS

**FIGURE 8:**
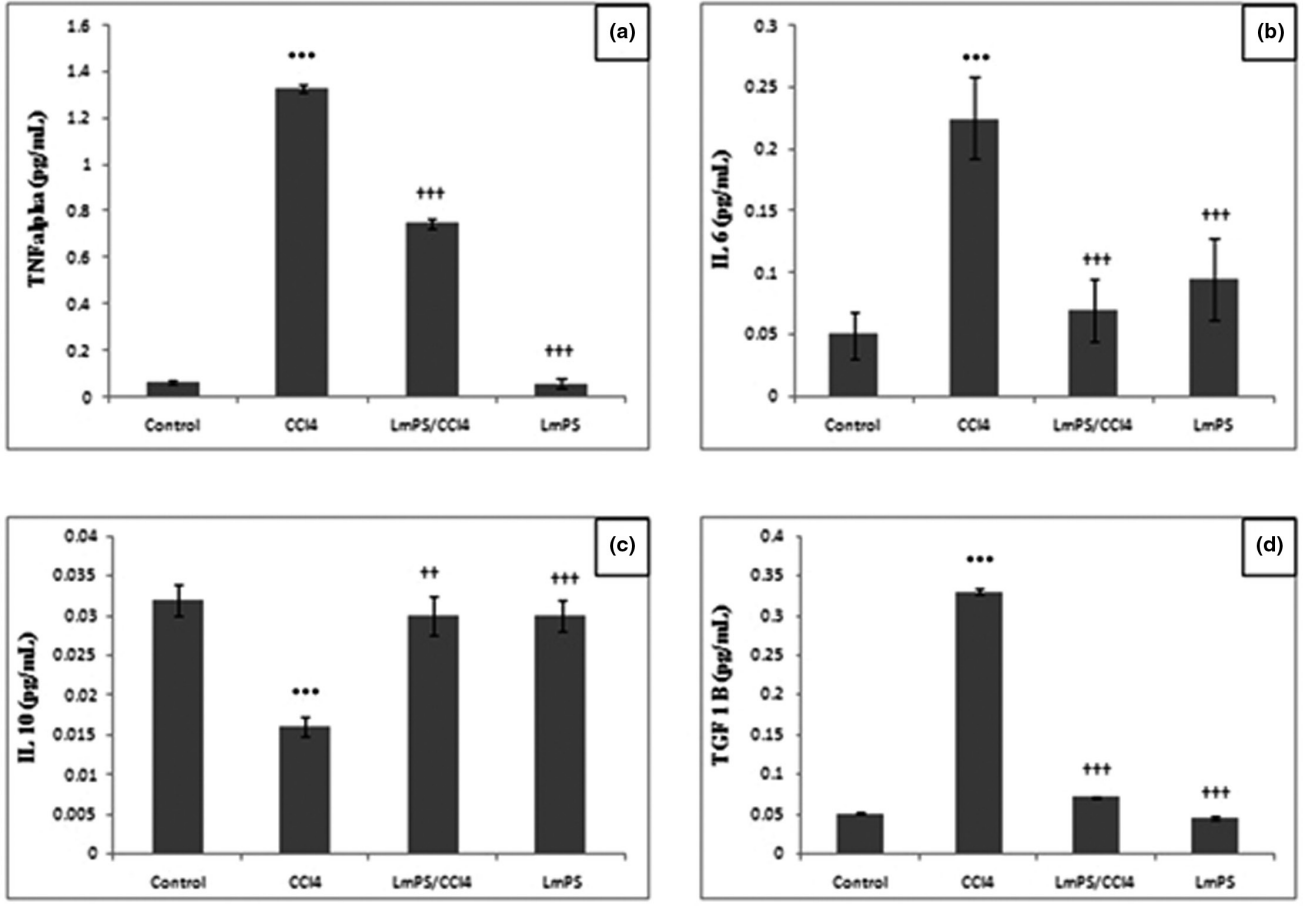
Protective and a therapeutic effect of LmPS on level of immune response in rats induced by CCl_4_ using commercially available kits

The inflammatory cytokine productions were evaluated in different treated groups and the results are summarized in Figure 8. As shown, there was a significant increase (*p* < .05) in TNF‐α (Figure 8a), IL‐6 (Figure 8b), and TGF‐β1 (Figure 8d) concentrations in liver tissues by reference to the control group due to CCl_4_. However, LmPS pretreatment (250 mg/kg) induced a significant decrease (*p* < .05) of these amounts. By comparison to control group, an important reduction of IL‐10 (Figure 8c) amount was found in animals treated by CCl_4_ alone. However, LmPS pretreatment in mixture with CCl_4_ caused a significant augmentation of IL‐10 level in hepatic tissues. As shown in Figure 8d, it was an important rise of serum TGF‐β1 amount in the CCl_4_ group (0.33 pg/ml) considering the control (0.05 pg/ml) group used as reference. In contrast, in animals receiving LmPS at a dose of 250 mg/kg and treated with CCl_4_, we noticed a substantial decrease of this parameter compared to CCl_4_ group. These results confirmed the anti‐inflammatory effects of LmPS marked by a small quantities of IL‐6, TNF‐α, and TGF‐β1 and high induction of anti‐inflammatory cytokine (IL‐10). Thus, this LmPS could be used as anti‐inflammatory agent.

### 3.11 DNA fragmentation

Results showed that CCl_4_ caused an important hepatic DNA fragmentation in hepatic tissues. We noted a rapid DNA migration and a long smear (data not shown) by reference to the control group DNA. LmPS pretreatment caused a significant decrease of the smear length; furthermore, we did not detect DNA fragments in the two animal groups (data not shown).

## 4 DISCUSSION

We were interested in the extraction, characterization, composition, and spectroscopic analysis of a novel heteropolysaccharide from Tunisian halophyte *L*. *maritima* (LmPS). LmPS was chemical studied using HPLC‐FID, UV‐visible spectroscopy, FTIR spectroscopy, TGA, HPLC, and TLC analysis; its potential health benefits as anti‐inflammatory agent have also been investigated. Results from this study proved that the LmPS amount was estimated at 8.5 ± 0.89 (w/w). This yield was better than other vegetables polysaccharides such as *Chaetomorpha antennina* (1.3%) (Jebamalar & Sumathy, 2018) and Chickpea (5.56%) (Mokni Ghribi et al., 2015). Temperature, time, environmental conditions, habitat, and water amount are reported to affect the polysaccharide extraction yields (Ben Slima et al., 2018). The total sugars accounted for 84.85 ± 0.56% of the extract. Similar findings were reported for *Sorghum* and *Opuntia ficus indica* cladodes polysaccharides (78.84% and 85.31%, respectively) (Bayar et al., 2016; Ben Slima et al., 2018). Protein content registered for our LmPS was 0.6% which considered as contaminants of cell wall polysaccharides (Yaich et al., 2013).

On the other hand, the ash and fat were estimated at 7.43% and 0.1%, respectively. These findings were similar to those previously reported for the polysaccharide extracted from *Sorghum bicola* (Ben Slima et al., 2018). Our sample had low moisture (7.02%). In addition, a high light value characterized the extracted LmPS (L* = 56.92) and a slight degree of redness (2.19) have been shown.

Our findings are in concordance with those reported by Ben Slima et al. (2018). According to them, *Sorghum* polysaccharides were characterized by a light color. The UV‐visible absorbance variations revealed a maximum value which confirmed the polysaccharide identification. This corroborates with previous results (Ben Slima et al., 2018; Hamzaoui et al., 2020; Trabelsi et al., 2018). LmPS broadly exhibited hydroxyl chemical groups (Wu, 2009). The peak region between 2881 and 2800 is associated to CH groups of LmPS free sugars (Zhu et al., 2013). In addition, absorption peaks between 800 and 1200 cm^−1^ attributed to carbohydrate fingerprints and functional groups LmPS specific to polysaccharides as stretching (C‐O‐C), bending (O‐H), and deforming (CH3) vibrations have been demonstrated (Trabelsi et al., 2009). The bands below 1000 cm may be due to visible bands and/or possible linkages between various monosaccharides (Parikh & Madamwar, 2006). A 844 cm^−1^ peak is due to α‐configuration in this polysaccharide (Ross et al., 2015).

Reactive oxygen species (ROS) are well‐reputed for their implication in heart diseases, diabetes, and cancer (AbouGabal et al., 2015). Normal cellular physiology requires a balanced state between ROS production and clearance (Droge, 2002). Antioxidants play a key role in the protection of cells against oxidative stress induced by excessive free radicals (Halliwell, 1995; Valko et al., 2007). However, some commercial synthetic antioxidants have harmful health effects (Bailey, 2005). Hence, natural antioxidants constitute an interesting alternative for synthetic ones. Free radical scavenging activities are attributed to several plant, algal, and fungal polysaccharides (Lima et al., 2016; Zhao et al., 2015). In our study, the antioxidant potential of LmPS crude polysaccharides was evaluated in vitro and in vivo.

Several methods use DPPH free radical to ascertain extract antioxidant potential (Ben Hsouna et al., 2013). LmPS DPPH scavenging activity was proportional to extract concentration. This finding is similar to those relative to many plant‐derived polysaccharides (Xu et al., 2009).

Furthermore, in this study, linoleic acid (C18:2) was oxidized in a water emulsion. LPO was inhibited by antioxidants which scavenge lipid‐derived radicals (Yin et al., 2011). In our study, LmPS had an important inhibiting potential against C18:2 peroxidation with a value closer to that of Catechin, at 100 µg/ml. Thus, it may be considered as an important antioxidant. This antioxidant property of LmPS may be attributed to numerous structural properties (Shahidi & Zhong, 2010; White et al., 2014).

The antioxidant property of a compound may be correlated with its reducing effect, which is mainly associated with reductones, able to interrupt free radical chain (Ben Hsouna et al., 2013).

According to our findings, LmPS possessed a moderate reducing power. This power is proportional to polysaccharide concentration. LmPS crude polysaccharide form is able to donate hydrogen, thus reacting with free radicals to stabilize and end up radical chain reactions. LmPS chelating effect may be associated to Fe^2+^ chelating groups. It is well established that there is no relationship between glucan and non‐glucan polymers antioxidant and intrachain linkages, the molecular weight, or the degree of polymer branching (Tsiapali et al., 2001).

In the hepatic parenchyma cells, CCl_4_ is transformed into trichloromethyl radicals (CCl_3_.) under cytochrome P450 action. Trichloromethylperoxyl radical (CCl_3_OO.) is the product of interaction between trichloromethyl radical and oxygen. This compound is able to cause damages to cellular membrane polyunsaturated fatty acids (PUFA) inducing a lack of membrane integrity and microsomal enzymes (AbouGabal et al., 2015; Ganapaty et al., 2013). Our results revealed, for the first time, that treatment with the crude polysaccharide LmPS could play a protective role against CCl_4_‐induced liver injury and genotoxicity in rats.

Crude LmPS significantly protected against CCl_4_‐induced liver damage through a decrease of AST, ALT, ALP, and LDH serum activities. An important activity of these enzymes is linked to a cellular leakage and loss of the integrity of the hepatocyte membrane (El‐Haskoury et al., 2018). The levels of these enzymes were stabilized by the *L. maritima* polysaccharide. LPO increased by CCl_4_ induced an increase in hepatic in treated animals. We noticed an increase of LPO as MDA amount in the treated group indicating a damaged status of hepatocytes as reported in previous works (Aromose et al., 2018; El‐Haskoury et al., 2018).

The crude LmPS was able to prevent increase of MDA content due to CCl_4_. This indicates an inhibition of LPO and protects hepatocytes from its propagation. This corroborates with the previous reports (Dasgupta et al., 2004).

The intracellular concentration of ROS depends not only on their production but also on their elimination by various antioxidants. In humans, three enzymes, namely SOD, CAT, and GPx, play a key role in cell protection against oxidative stress. A synergic activity characterizes these enzymes required into superoxide anion and H_2_O_2_ cell detoxification.

We noticed an increase of antioxidant enzymes activity consecutive to the pretreatment with *L*. *maritima* crude polysaccharide. Endogen enzymes protect cells against oxidative stress damages. We were interested in LmPS corrective effect on oxidative stress induced by CCl_4_. This proved that LmPS administration at 250 mg/kg exhibited hepatoprotective effect. Considering these findings, *L*. *maritima* could be a valuable source of natural compounds with interesting therapeutic properties.

Inflammation is associated to hepatic fibrogenesis in chronic liver diseases (Seki & Schwabe, 2015). Since LmPS is able to inhibit liver inflammation and to enhance IL‐10 secretion, it can protect the liver from CCl_4_ harmful effects. According to our findings, there was an increase of serum TNF‐α concentration in the CCl_4_ group by reference to the control one. Furthermore, in this study, we noted a CCl_4_ infiltration of inflammatory cells in liver. Ezz et al. (2011) found similar results. According to these authors, TNF‐α amount increases due to CCl_4_ administration. Kupffer cells simulated by CCl_4_ secrete many cytokines, among them TNF‐α. At high amounts, cytokines are capable of improving the immune response to double speed which results in liver cirrhosis (Li et al., 2001).

According to several previous results, IL‐10 was proven to have antifibrotic, anti‐inflammatory, and immunomodulatory effects. TGF‐β1 cytokine plays a key role in liver fibrosis (Fan et al., 2013). We noted an important increase of TGF‐β1 serum level in the group treated with CCl_4_. Similar findings were reported by Hafez et al. (2017).

CCl_4_ is actively required in the initiation of liver necrosis. It improves the secretion of factors implicated in fibrogenesis like TGF‐β1 and TNF‐α. Besides, LmPS significantly caused in vivo a decrease of TGF‐β1 levels at doses 250 mg/kg due to CCl_4_. An increase of the TGF‐β1 activation in experimental rats administered by a single dose of CCl_4_ has been reported (Chang et al., 2007; Jiang et al., 2004). According to the same authors, this effect was reversed by a plant administration *L. maritima*. These results confirm those of Kohli et al. (2005) who reported antifibrogenic properties for IL‐10 mediated through a down‐regulated secretion of TGF‐β1 and TNF‐α. In the same context, Shi et al. (2014) reported an inhibition of fibrosis by IL‐10.

Important massive fatty changes were detected in the liver of CCl_4_‐intoxicated rat group. The group pretreated with purified LmPS and subsequently given CCl_4_ was characterized by a normal histology.

In the addition to the oxidative stress, CCl_4_ may induce genotoxicity (Sahreen et al., 2011). CCl_4_‐induced LPO concerns proteins and also DNA (Bhadauria & Nirala, 2009; Khan et al., 2012). In fact, MDA reacts with the DNA strand to form the MIG adduct, the mutagenic pyrimidopurinone adduct of deoxyguanosine (Chaudhary et al., 1996). We were interested in LmPS eventual preventive effect against DNA fragmentation due to CCl_4_ administration. Integrity of hepatic DNA was preserved by LmPS pretreatment of CCl_4_‐intoxicated group. Altogether, our results clearly evidenced the antigenotoxic capacity of this LmPS polysaccharide. LmPS protective effect against CCl_4_‐induced genotoxicity may be explained by the fact that it is able to inhibit CCl_4_ oxidative process. Similar findings were reported by Nagwani and Tripathi (2010). Considering all these findings, a significant effect of LmPS on CCl_4_‐induced hepatotoxicity, oxidative stress, and genotoxicity was evidenced for the first time as anti‐inflammatory agent.

## 5 CONCLUSION

According to the findings of our study, LmPS effectively protects rats against CCL4 induced hepatotoxicity in vivo. This heteropolysaccharide is composed of glucose, galactose, and xylose determined by TLC and HPLC. This polysaccharide induced higher anti‐inflammatory cytokines and small quantities of proinflammatory cytokines. According to in vitro studies, LmPS protective potential could be correlated to its high antioxidant activities. For this reason, further studies are currently under way to fully better understand the in vivo mechanism of this polysaccharide.

## CONFLICT OF INTEREST

The authors declare that they have no conflict of interest.

## Data Availability

Data available on request by the first author, Anis Ben Hsouna (benhsounanis@yahoo.fr).
